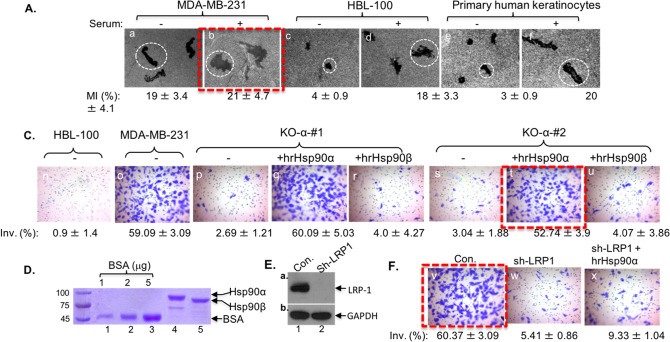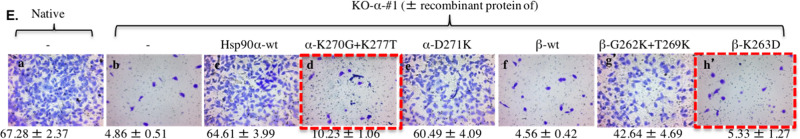# Correction: Evolutionarily conserved dual lysine motif determines the non-chaperone function of secreted Hsp90alpha in tumour progression

**DOI:** 10.1038/s41388-024-03017-0

**Published:** 2024-04-04

**Authors:** M. Zou, A. Bhatia, H. Dong, P. Jayaprakash, J. Guo, D. Sahu, Y. Hou, F. Tsen, C. Tong, K. O’Brien, A. J. Situ, T. Schmidt, M. Chen, Q. Ying, T. S. Ulmer, D. T. Woodley, W. Li

**Affiliations:** 1 Department of Dermatology and the Norris Comprehensive Cancer Center, Los Angeles, CA USA; 2 Eli and Edythe Broad Center for Regenerative Medicine and Stem Cell Research and Department of Cell and Neurobiology, Los Angeles, CA USA; 3 Department of Biochemistry and Molecular Biology and Zilkha Neurogenetic Institute University of Southern California Keck Medical Center, Los Angeles, CA USA; 4 Department of Medical Research, Greater Los Angeles Veterans Affairs Heath Care System, Los Angeles, CA USA; 5 Present Address: Department of Endocrinology and Metabolism, and Department of Respiratory and Critical Care Medicine, Chronic Airways Diseases Laboratory, Nanfang Hospital, Southern Medical University, Guangzhou, 510515 China

Correction to: *Oncogene* 10.1038/onc.2016.375, published online 10 October 2016

Following the publication of this article, it was noted that the positive control of MDA-MB-231 cells in Figure 2F (panel v) and a non-specific mutant control of recombinant Hsp90β in Figure 4E (panel h) were mistakenly duplicated with the images in Figure 2C (panel t) and in Figure 4E (panel d), respectively. The positive control of MDA-MB-231 cells in Figure 2A (panel b) was also a duplicate of Figure 4C (panel b) in [1].

Figures 2A, 2F, and 4E have now been corrected as shown below.

The authors confirm these amendments have no impact on the results presented in this article and apologize for any inconvenience caused.